# Evaluation of the Reproducibility and Robustness of Extrusion-Based Bioprinting Processes Applying a Flow Sensor

**DOI:** 10.3389/fbioe.2022.831350

**Published:** 2022-03-03

**Authors:** Svenja Strauß, Bianca Schroth, Jürgen Hubbuch

**Affiliations:** ^1^ Karlsruhe Institute of Technology, Institute of Functional Interfaces, Karlsruhe, Germany; ^2^ Institute of Engineering in Life Sciences, Section IV: Molecular Separation Engineering, Karlsruhe Institute of Technology, Karlsruhe, Germany

**Keywords:** flow rate control, flow rate measurement, reproducibility, bioprinting, (printing) process control

## Abstract

Bioprinting is increasingly regarded as a suitable additive manufacturing method in biopharmaceutical process development and formulation. In order to manage the leap from research to industrial application, higher levels of reproducibility and a standardized bioprinting process are prerequisites. This said, the concept of process analytical technologies, standard in the biopharmaceutical industry, is still at its very early steps. To date most extrusion-based printing processes are controlled over penumatic pressure and thus not adaptive to environmental or system related changes over several experimental runs. A constant set pressure applied over a number of runs, might lead to variations in flow rate and thus to unreliable printed constructs. With this in mind, the simple question arises whether a printing process based on a set flow rate could improve reproduciblity and transfer to different printing systems. The control and monitoring of flow rate aim to introduce the concept of PAT in the field of bioprinting. This study investigates the effect of different processing modes (set pressure vs. set flow rate) on printing reproducibility occurring during an extrusion-based printing process consisting of 6 experimental runs consisting of 3 printed samples each. Additionally, the influence of different filling levels of the ink containing cartridge during a printing process was determined. Different solutions based on a varying amount of alginate polymer and Kolliphor hydrogels in varying concentrations showed the need for individual setting of printing parameter. To investigate parameter transferability among different devices two different printers were used and the flow was monitored using a flow sensor attached to the printing unit. It could be demonstrated that a set flow rate controlled printing process improved accuracy and the filling level also affects the accuracy of printing, the magnitude of this effects varies as the cartridge level declined. The transferability between printed devices was eased by setting the printing parameters according to a set flow rate of each bioink disregarding the value of the set pressure. Finally, by a bioprinting porcess control based on a set flow rate, the coefficient of variance for printed objects could be reduced from 0.2 to 0.02 for 10% (w/v) alginate polymer solutions.

## 1 Introduction

In the fields of regenerative medicine (RM) and tissue engineering (TE), the precise manufacturing of unique and artificial tissues is the key element enabling the development towards personalized medicine (Atala, 2009; Murphy and Atala, 2014a; Mao and Mooney, 2015; Groll et al., 2016). These systems can be used for the replacement of damaged tissues or as drug delivery systems. Moreover, they can facilitate and standardize clinical or pharmaceutical studies (Caddeo et al., 2017; Culver et al., 2017; Chen et al., 2020). 3D bioprinting as an advanced additive manufacturing method opens up the possibility to build complex tissue constructs by applying a bioink, which usually consists of a hydrogel cell mixture, in layers with spatial control (Malda et al., 2013; Groll et al., 2018). Hydrogels are suitable for engineering bioinks as they closely resemble natural tissues, offer mild conditions for cells or biological materials, and are biocompatible (Hoffman, 2012; Naahidi et al., 2017). Depending on the specific chemical and mechanical requirements for each artificial tissue, different hydrogels with varying modifications are employed (Smeds and Grinstaff, 2001; Yue et al., 2015). Much research has been done on the engineering of bioinks and companies already offer prepackaged bioinks commercially. However, bioinks are currently sold only for research purposes and not for clinical applications. In this study Kolliphor or also called poloxamer is used as it is a synthetic model hydrogel which is partly employed to establish new methods and as sacrificial material in the bioprinting field (Paxton et al., 2017; Ribeiro et al., 2017; Radtke et al., 2018). Natural alginate solutions were also used, as alginate, with the advantages of a natural polymer and its viscous properties, is often the basis for bioink formulations (Rowley et al., 1999; Axpe and Oyen, 2016). In terms of process engineering or process development within the field of 3D bioprinting, hydrogels and their characteristics are the dominant factor being the carrrier of biological material. As hydrogels are viscoelastic materials which combine the characteristics of elastic solids and Newtonian fluids, the success of an extrusion process is strongly influenced by the rheological properties of the bioink (Paxton et al., 2017; Ribeiro et al., 2017; Bonatti et al., 2021). The viscosity is highly temperature-sensitive, and is further influenced by process parameters such as polymer concentration, pH, ionic strength, environmental pressure and UV radiation for UV-responsive polymers (Zhao et al., 2015; Jungst et al., 2016; Mezger, 2016). The yield point is also dependent on the material and represents the stress level at which the material starts to flow, meaning that the elastic behaviour turns into a plastic one (Osswald and Rudolph, 2014). A perfectly controlled environment would actually be needed to take all of this into account, but in reality fluctuating temperatures and humidity levels are usual. Additional problems arise through often observed inhomogeneities in the polymer solution occuring as a function of time and temperature, leading in some cases to nozzle clogging (Fisch et al., 1101; Seiffert and Sprakel, 2012). When working with materials from natural sources, the batch-to-batch variance must be taken into account and also the filling level within the cartridge might require an extrusion pressure adaptation. In order to counteract these challenges, bioprinters are developed within an atmospheric enclosure system for controlling the environmental conditions such as temperature, humidity, and carbon dioxide concentration (Matamoros et al., 2020). Such systems are certainly expensive and cannot react to material changes. Therefore, cheaper and more general solutions are needed that can react to environmental and material changes.

**FIGURE 1:**
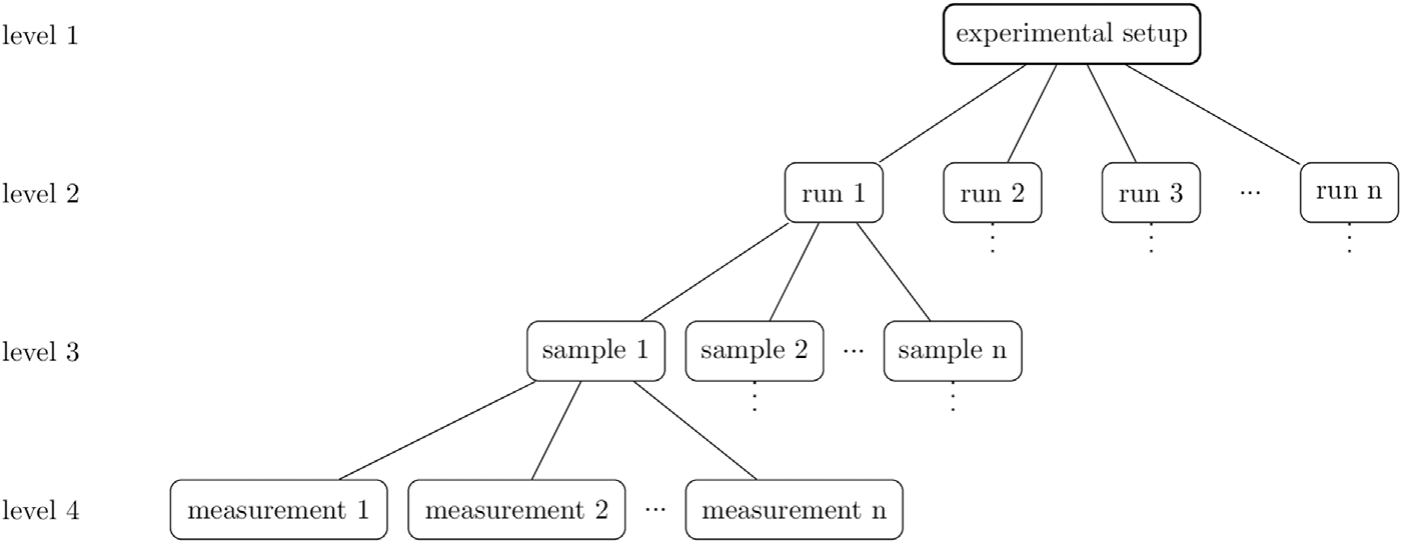
A hierarchical overview of the experiment design. Level 1 includes the experimental setup, which for 3D bioprinting is represented, for example, by the bioprinter used, the printing parameters, and the bioink. Level 2 consists of biological replicates, the runs. Several objects can be printed in one run which are the samples (level 3). These samples are technical replicates of the printing process and can be analyzed in a further step. The result is a measurement and in the case of a multiple determination, these measurements are also technical replicates of the analysis method.

Currently, printing parameters are determined during a printing process setup and thus kept constant during several runs (Figure 1, level 1). However, as described above, it is important to react to environmental and material changes. Instead of tackling each of these issues individually, a more general and effective approach may be to define an optimal flow rate which is adjusted during the printing process. A bioprinting system developed by Philipp Fisch et al. using a progressive cavity pump which controls the volume flow by displacement already showed an improved printing accuracy, when compared to a constant pressure approach (Fisch et al., 1101). In order to set a desired flow rate for a conventional pneumatic system, the pressure could be adjusted based on the feedback of a flow sensor. Handling changes in temperature or cartridge fill level during the print would require a dynamic pressure adaptation in real-time (Fisch et al., 1101). To do so, a necessary requirement would be to equip each bioprinter with a mass flow sensor which regulates the mass flow e.g. via pressure in real time. Mass or flow rates can be determined with sensors based on mechanical, ultrasonic, electrical, or thermal methods (Rasmussen and Zaghloul, 1999). Flow sensors are widespread in the automotive industry, but are becoming more prevalent in the medical field where they are used for the controlled administration of infusions to patients (Nguyen, 1997; Fleming, 2001). Here, sensors with thermal measuring principle are established which are only in indirect contact with the medium and work under sterile conditions (Schnell and Schäfer, 1995). However, it is to be evaluated if such a process control during each sample is needed or if the adjustment before each run is enough (see Figure 1). To date, flow rates are realized by syringe pumps or using mechanical extrusion systems and flow rates are determined by weighing extruded material. A poster was published for a project in which a flow sensor was used to measure the flow rate. The results are not completely congruent with the weighed flow rates and, in general, there is currently a lack of flow sensors that are suitable for visco-elastic materials (Banović Vihar, 2018; Yan et al., 2012; Opel et al., et al., 2017).

Following this line of argumentation, this study revolves around the hypothesis that the reproducibility of bioprinting processes based on pneumatic extrusion can be improved by choosing a flow rate controlled process mode over a pressure controlled process mode. In order to valuate the effect of this change, a flow sensor is incorporated in an extrusion-based bioprinter and similar samples are printed and compared in two different process modes. In the first case, the samples are printed with the same extrusion pressure (set pressure, cP) determined in the experimental setup (Figure 1, level 1), and in the second case, a constant flow rate (set flow rate, cFR) is set by adapting the pressure of the printer system prior to each run (Figure 1, level 2). For this purpose, the flow sensor was used for initial calibration of the pressure required for the desired flow rate. In order to analyze the effect of filling level in the cartridges used (and thus need for a more dynamic control), the flow sensor is used to measure the flow rate for constant printing parameters during the complete emptying of one printer cartridge to examine the influence of the cartridge filling level. The investigation was carried out using two different extrusion printing systems and two inks with varying concentrations of Kolliphor and alginate.

## 2 Materials and Methods

In general, when analyzing 3D bioprints, the experimental design is important and influences the conclusions drawn. A clear distinction should be made between hierarchical levels but also between the types of replicates performed. An overview graphic of the hierarchical order is shown in Figure 1.

Right at the top (level 1) is the experimental setup, which for 3D bioprinting is represented, for example, by the printer used, the printing parameters, and the bioink. For the course of this paper we define the term “bioink” as an ink containing cellular material, while we use the term “ink” for a hydrogel or polymer solution without any additional biological material. The level below or level 2 consists of biological replicates, the runs. Biological replicates “are parallel measurements of biologically distinct samples, which may be random biological variation that is itself the subject of the study or a source of noise source” (Blainey et al., 2014). Transferred to bioprinting this means the independent production of bioink batches according to the same method. This said, this paper also speaks of biological replicates when the ink is produced without cells. Level 3 consists of samples when several objects are printed from the same bioink production. Thus, one sample is a technical replicate and if the samples are measured several times, the results are measurements, which are also technical replicates.

### 2.1 Ink Preparation and Printing Systems

For both printer systems, cartridges including pistons were obtained from Nordson Corporation (Westlake, United States), and plastic, conical 25 G nozzles with an inner diameter of 250 µm were ordered from Cellink (Gothenburg, Sweden) which were used for all experiments. Sodium alginate and Kolliphor P 407 were both obtained from Sigma Aldrich (St. Louis, United States) and were used for ink manufacturing in respective concentrations. The appropriate amount of powder for each ink was dissolved in ultrapure water (arium^®^ pro VF, Satorius AG, Göttingen, Germany). During filling the cartridges, attention was closely paid to a uniform distribution. Since the inks are not pipettable, 3 ml of water was first poured into the cartridges as a reference and the level was marked. Then, the cartridges were filled up to the optical mark while ensuring a uniform distribution without air bubbles. The inks were made no more than 12 h prior to filling and were stored in the refrigarator at 4°C. The samples were taken out of the refrigerator 15 min prior to each experiment. Each trial was carried out at room temperature.

The comparison of both process modes, namely cP and cFR, were performed with a pneumatic extrusion-based bioprinter 3D Discovery™ provided by regenHU company (Villaz-St-Pierre, Switzerland). The BioCAD software (regenHU, Villaz-St-Pierre, Switzerland) was used to create the CAD model and G-Code for printed objects. Additionally, filling level experiments were done with a BIO X bioprinter and the BIO X software v.1.8.1 was used (Cellink, Gothenburg, Sweden).

### 2.2 Density Calibration

With regard to a gravimetric verification of the flow sensor data, the densities for sodium alginate (0.25, 0.5, 0.75, 1, 1.5, 2, 2.5, 3% (w/v) concentration) and Kolliphor (1, 2, 3, 5, 10, 15% (w/v) concentration) were measured for 10 s using a micro liquid density sensor (ISSYS, Ypsilanti, United States). All measurements were performed in triplicates (*n* = 3), and the densities for higher concentrations were calculated with the straight line equation, since higher densities are no longer within the measurement range of the ISSYS.

### 2.3 SLI Liquid Flow Meter

**FIGURE 2:**
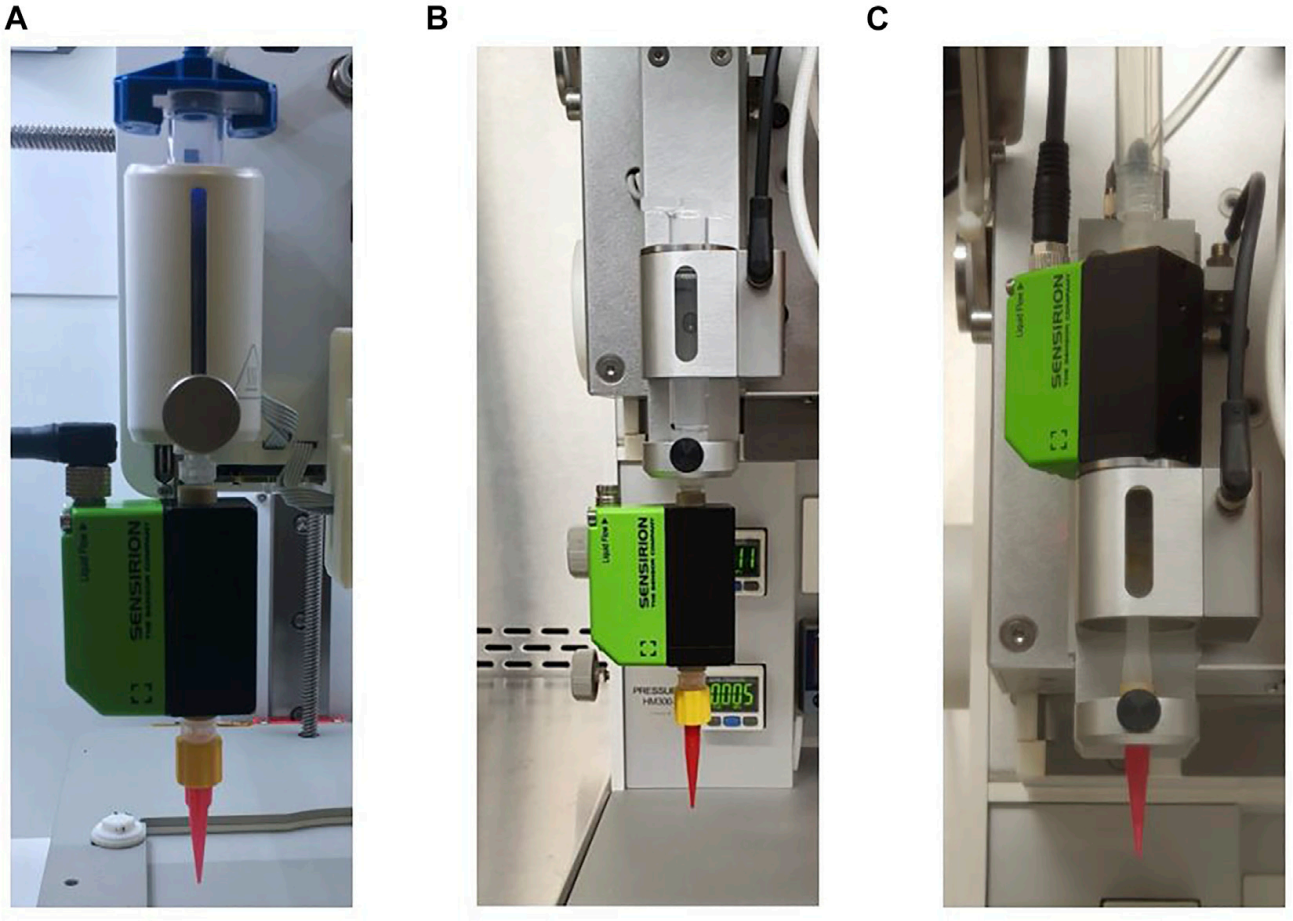
Different incorporation setups for the flow sensor into the bioprinters. For the investigation of cartridge filling level influence it was attached to BIO X as shown in **(A)** and to 3D Discovery™ as shown in **(B)**. The reprodicibility experiments were only performed with the 3D Discovery™ and a different attachment of the sensor depictured in **(C)**.

In this study, a flow sensor SLI-1000 FMK obtained from Sensirion (Staefa, Switzerland) was attached which is suitable for measurements of up to 10 ml min^−1^. Figure 2 shows how the sensor was incorporated for the investigation of cartridge filling level influence into BIO X (A) and into 3D Discovery™ (B). The reprodicibility experiments were only performed with the 3D Discovery™ and a different attachment of the sensor depictured in (C).

**FIGURE 3:**
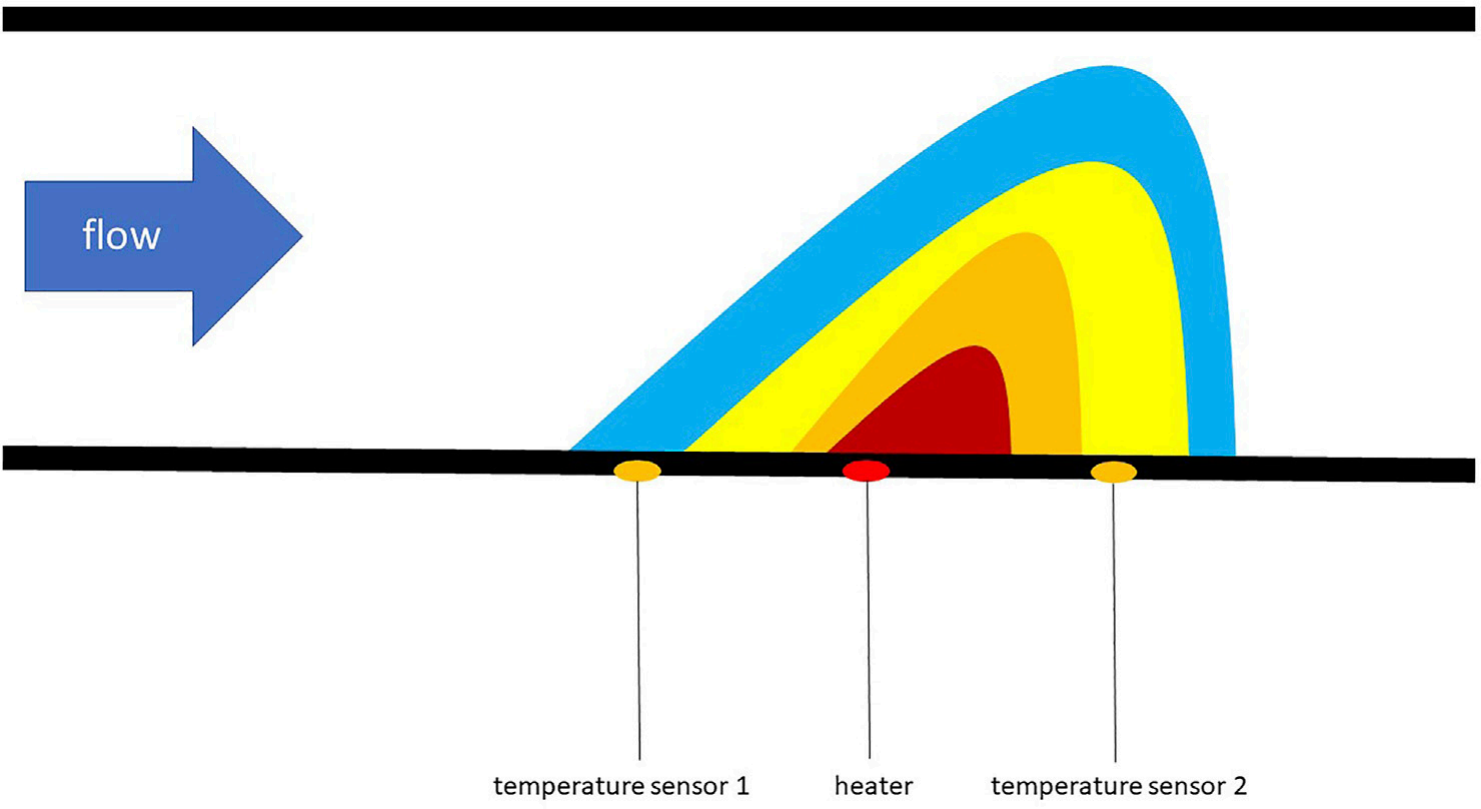
Illustration of the thermal based measuring principle of the flow sensor adapted from Kuo (Kuo et al., 2012).

Inside there is a borosilicate glass capillary with an internal diameter of 1 mm and a wall thickness of 100 µm. The total internal capillary volume is 25 µL and the capillary was prefilled before each experiment. The flow rate is determined using a thermally based measuring principle, which is shown in Figure 3. It consists of a heating element between two temperature sensors on the outside of the capillary and the flow rate is calculated in the software using the temperature difference between the temperature sensors. The liquid is never in direct contact with the measuring chip and the detection delay is 40 ms.

#### 2.3.1 Flow Sensor Calibration

Since the sensor was originally developed for liquids, the applicability for the selected inks first had to be verified and calibrated for the respective inks. This was done for ink containing alginate with the concentrations of 8, 10, 12, and 15% (w/v) and for ink containing Kolliphor with 15, 20, 25, 28, and 30% (w/v). The sensor was connected via Luer lock to a syringe pump neMESYS (Cetoni GmbH, Korbußen, Germany). For each concentration of alginate and Kolliphor, a rough screening was carried out beforehand to ensure that the maximum adjustable speed of the pump was still in the measuring range of the sensor. Thereafter, at least seven flow rates for every concentration were measured in triplicates (*n* = 3).

#### 2.3.2 Flow Sensor Accuracy

After calibration, measurement accuracy was analyzed and defined as the deviation between cylinder volumes calculated based on sensor data and cylinder volumes based on weighed values:

$$Deviation=\frac{V_{Q}}{V_{m}}$$ (1)

*V*_*Q*_ is here the cylinder volume in µL calculated with the sensor data using the following Eq. 2 where $\dot{Q}$ is the flow rate in µLmin^−1^ measured by the sensor:

$$V_{Q}=\int \dot{Q}\;dt$$ (2)

*V*_*m*_ is the volume in µL for each cylinder based on the weighed mass *m* in g divided by the density *ρ* in g cm^−3^. Using the density determined as described in Section 2.2, the volume for each cylinder could be calculated according to Eq. 3:

$$V_{m}=\frac{m}{\rho }$$ (3)

To determine the accuracy, five hollow cylinders (*n*_*sample*_ = 5) each with a diameter of 10 mm and 15 layers with a 0.33 mm layer height were printed and weighed immediately after printing using an analytical balance AB204-S obtained from Mettler-Toledo GmbH (Gießen, Germany). This was done for alginate concentrations of 8, 10, 12, and 15% (w/v) and for Kolliphor concentrations of 15, 20, 25, 28, and 30% (w/v). The printing parameters are listed in Table 1 and were defined in a print optimization with the aim of printing intact hollow cylinders.

**TABLE 1:** Printing parameters for testing the sensor accuracy with inks of different alginate and Kolliphor concentrations. Z offset is the distance between nozzle and substrate when the first layer is printed.

Concentration	Ink_*Kolliphor*_					Ink_*Alginate*_			
[% (w/v)]	15	20	25	28	30	8	10	12	15
Pressure [MPa]	0.01	0.105	0.195	0.22	0.36	0.06	0.08	0.12	0.27
Speed [mm/s]	10	10	10	10	20	20	20	20	20
Height [mm]	0.33	0.33	0.33	0.33	0.33	0.33	0.33	0.33	0.33
Z offset [mm]	0.05	0.05	0.05	0.05	0.05	0.05	0.05	0.05	0.05

### 2.4 Investigation of Cartridge Filling Level Influence

To obtain an overview of whether the filling level within a cartridge influences the bioprinting process, cartridges were filled with maximal filling level of 3 ml of ink containing Kolliphor concentrations of 15, 20, and 25% (w/v) and with alginate concentrations of 10, 12, and 15% (w/v) were dispensed at a constant pressure (see Table 2) until only air was extruded. This was repeated for three runs (*n*_*run*_ = 3) on both bioprinters, BIO X and 3D Discovery™. A summary of all setups is shown in Table 3. The flow rate was monitored over the entire period using the flow sensor which was incorporated into the bioprinters as presented in Figures 2A,B.

**TABLE 2:** Set pressures applied during filling level investigations for the ink with the respective alginate and Kolliphor concentration.

Concentration	Ink_*Kolliphor*_			Ink_*Alginate*_			
[% (w/v)]	15	20	25	8	10	12	15
Pressure [MPa]	0.015	0.105	0.195	0.05	0.1	0.15	0.195

**TABLE 3:** Summary of all setups for the investigation of cartridge filling level influence. Ink_*Alginate*_ is an abbreviation for all tested alginate concentrations of 8, 10, 12, and 15% (w/v) and Ink_*Kolliphor*_ for all tested Kolliphor concentrations of 15, 20, 25, 28, and 30% (w/v). Process mode means either constant pressure (cP) or constant flow rate (cFR).

Setup			No. of runs	No. of samples
Bioprinter	Ink	Process Mode		
BIO X	Ink_*Alginate*_	cP	3	1
3D Discovery™	Ink_*Alginate*_	cP	3	1
BIO X	Ink_*Kolliphor*_	cP	3	1
3D Discovery™	Ink_*Kolliphor*_	cP	3	1

### 2.5 Reproducibility Experiments

In order to investigate the reproducibility of the bioprinter, again a hollow cylinder with a diameter of 10 mm and 15 layers with 0.33 mm layer height was printed. In the experimental setup, the 3D Discovery™ was used (Figure 1, level 1) and the flow sensor was attached as presented in Figure 2C. A summary of all setups is shown in Table 4. In total 6 runs printing ink containing Kolliphor with a concentration of 25, 28, and 30% (w/v) and ink containing alginate with a concentration of 10, 12 and 15% (w/v) were performed (Figure 1, level 2). For each run, 3 cartridges were filled with 3 ml of one batch and one sample printed from one cartridge (Figure 1, level 3). We thus carried out 6 biological replicates (*n*_*run*_ = 6, level 2) and for each of those 3 technical replicates (*n*_*sample*_ = 3, level 3). Thus in total 18 cylinders were printed for each ink composition.

**TABLE 4:** Summary of all setups for the reproducibility experiments. Ink_*Alginate*_ is an abbreviation for all tested alginate concentrations of 10, 12, and 15% (w/v) and Ink_*Kolliphor*_ for all tested Kolliphor concentrations of 25, 28, and 30% (w/v). Process mode means either constant pressure (cP) or constant flow rate (cFR).

Setup			No. of runs	No. of samples
Bioprinter	Ink	Process Mode		
3D Discovery™	Ink_*Alginate*_	cP	6	3
3D Discovery™	Ink_*Alginate*_	cFR	6	3
3D Discovery™	Ink_*Kolliphor*_	cP	6	3
3D Discovery™	Ink_*Kolliphor*_	cFR	6	3

In the experimental setup (level 1) it was decided to compare two modes of processing. In the first case, a constant pressure (cP) for all runs was applied and in the second case, the pressure was adjusted to set a constant flow rate (cFR) for all runs. The latter was achieved by manually adjusting the pressure prior to each run until the desired flow rate was set. The respective printing parameters and flow rate target settings of all inks are listed in Table 5. In general, several printing parameter combinations can be selected for a printing process in order to achieve the same result. If the speed is increased, the pressure must also be increased. Because different speeds were set for the combinations in a screening before the study, the pressure does not increase with increasing concentration. The aim of the screening was to be able to print intact cylinders.

**TABLE 5:** Printing parameters for the investigation of reproducibility. The set flow rate values for the cFR case (last line) correspond to the sensor data and not to the actual flow rates. The Z offset means the distance between nozzle and substrate when the first layer is printed.

Concentration	Ink_*Kolliphor*_			Ink_*Alginate*_		
[% (w/v)]	15	20	25	10	12	15
Pressure [MPa]	0.3	0.22	0.36	0.08	0.12	0.27
Speed [mm/s]	15	10	20	20	20	20
Layer height [mm]	0.33	0.33	0.33	0.33	0.33	0.33
Z offset [mm]	0.05	0.05	0.05	0.05	0.05	0.05
Flow rate [µl/min]	7,636.2	6,323.4	6,617	1,500.4	1,645.2	1941.7

### 2.6 Data Analysis

Data evaluation, statistical data analysis, and visualization were done with Matlab^®^ R2019a (TheMathWorks, Natick, United States). Statistical analysis was performed with the calculated cylinder volumes of the reproducibility experiments. Since the normal distribution check using the Anderson-Darling test did not result in a normal distribution for all data sets, Mann Whitney U test as non-parabolic test was chosen. It was used to compare the two data sets of the two different process strategies cP and cFR. This was done for each of the six evaluated inks containing different concentrations of Kolliphor and alginate. For these investigations, *α* was set to 0.1 and a *p*-value below 0.05 was classified as statistically significant. Statistical significance is marked by an asterix in the figure.

## 3 Results

### 3.1 Sensor Calibration

In preparation to use the sensor for ink measurements, a calibration for all inks containing different concentrations of Kolliphor and alginate was performed. This was done for each concentration with at least five flow rates in triplicates (*n* = 3) using a syringe pump. All data sets can be found in the Supplementary Data.

### 3.2 Printing Accuracy

Accuracy is a measure of a deviation between an obtained object or measurement performed and its theoretical model/value. The volumetric deviation obtained by determining the applied volume gravimetrically and the calculated volume by using Eq. 1. The density for the specific concentrations was also measured in triplicates (*n* = 3) in order to convert the volumetric flow rates into mass flows and data are shown in the Supplementary Data.

**FIGURE 4:**
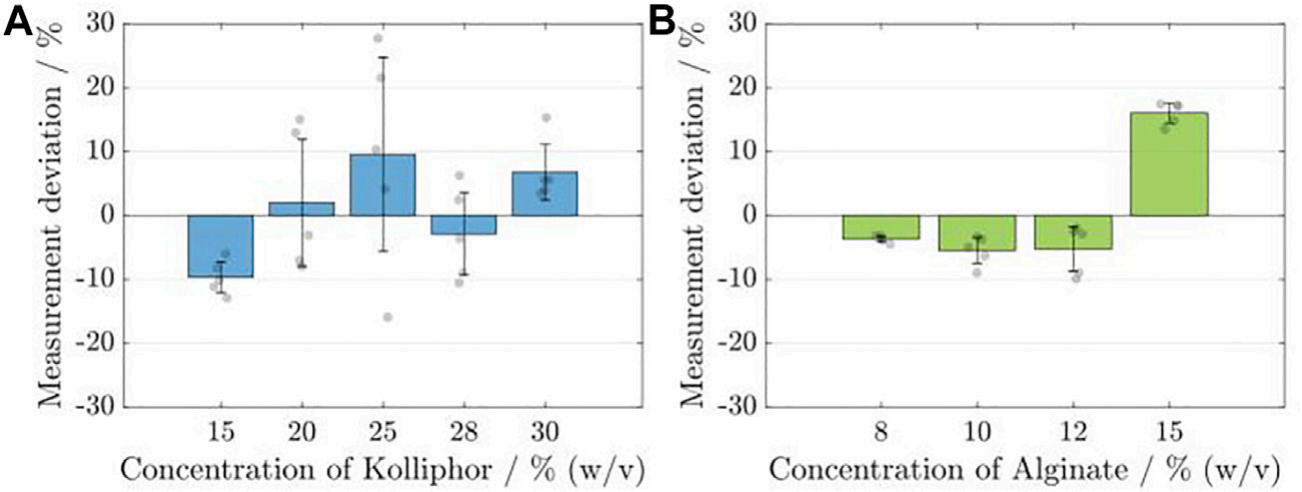
Analysis of the calibration and measurement accuracy of the flow sensor for the respective Kolliphor **(A)** and alginate **(B)** concentrations. Each time, five identical cylinders (*n*_*sample*_ = 5) were printed, and the calculated volumes based on the sensor data were compared with the data from the gravimetric determination. The deviation of the sensor measurement is given in percent.

The same hollow model cylinder was printed five times (*n*_*sample*_ = 5) with identical model and printing parameters in a single run. The respective deviation of the sensor data from the gravimetrically determined data is shown for the Ink_Kolliphor_, applying different concentrations in Figure 4A and for Ink_Alginate_, applying different concentrations in Figure 4B.

For none of the Kolliphor concentrations, the mean deviation obtained for the five samples is higher than 10%, and the maximum deviation was found to be −9.66 ± 2.39% for a 15% (w/v) concentration. The smallest deviation is 1.99 ± 9.97% for a 20% (w/v) Kolliphor solution. The standard deviation is highest for ink containing 25% (w/v) Kolliphor at 15.19% and lowest at 2.39% for ink containing 15% (w/v) Kolliphor. For alginate containing inks, the maximum deviation is 16.06 ± 1.58% for a 15% (w/v) alginate solution and the smallest is at −3.65 ± 0.49% for a concentration of 8% (w/v) alginate solution. In comparison to Kolliphor containing inks, the standard deviation obtained with the alginate containing inks is lower by a factor of about 5 with a maximum of 3.46% for ink containing 12% (w/v) alginate and a minimum of 0.49% for 8% (w/v) alginate solution. For alginate containing inks, it can be stated that the sensor’s measurement accuracy decreases with rising alginate concentration and the associated increase in viscosity. For Kolliphor containing inks a random distribution was obtained.

### 3.3 Influence of Cartridge Filling Level

**FIGURE 5:**
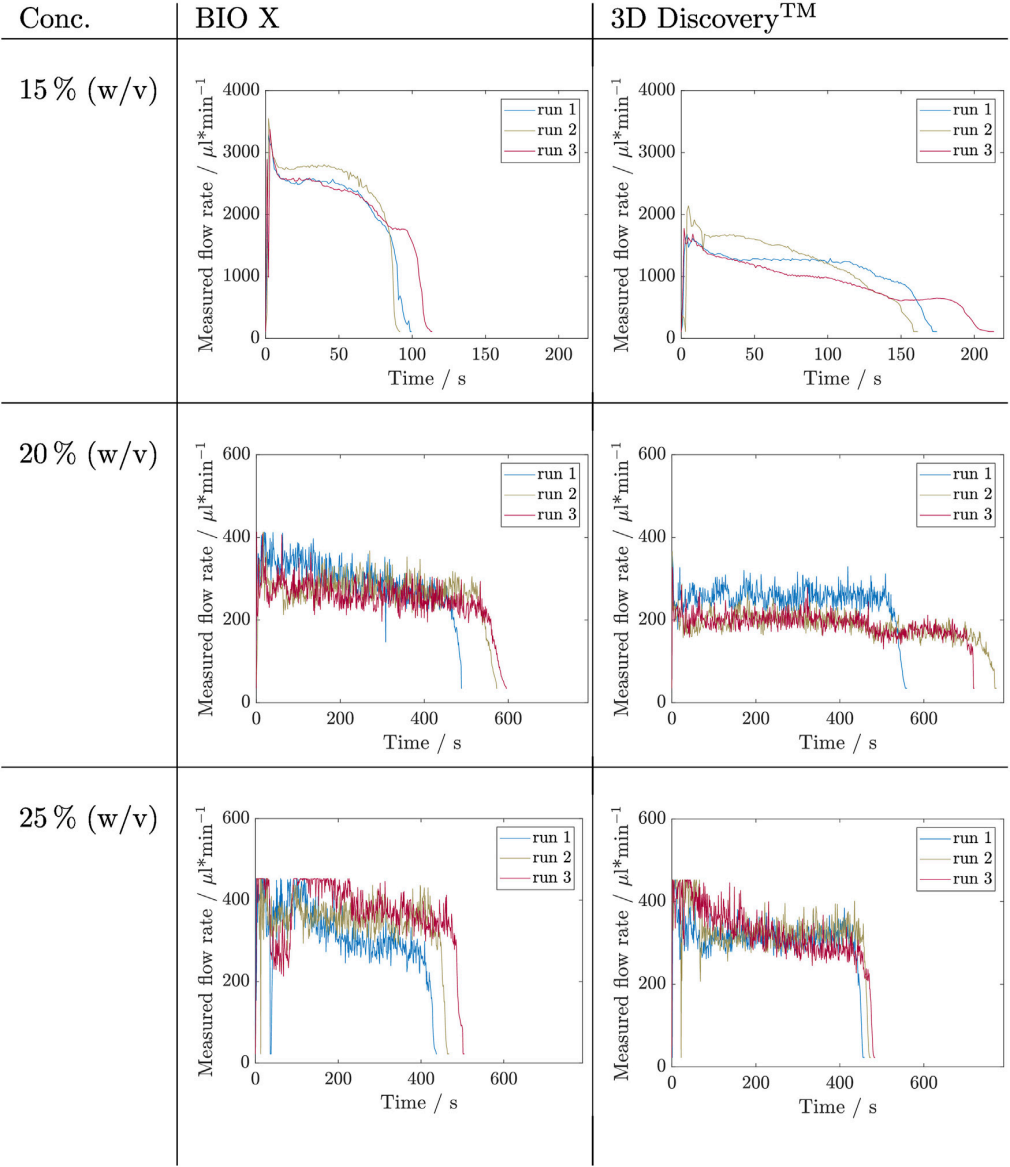
Results of filling level influence investigation for inks containing Kolliphor concentrations of 15, 20, and 25% (w/v) by measuring the flow rate during the complete emptying of a cartridge. This experiment was carried out in triplicates (*n*_*run*_ = 3) for each concentration and on two bioprinter systems, namely BIO X and 3D Discovery™. The respective set pressures are listed in Table 3.

**FIGURE 6:**
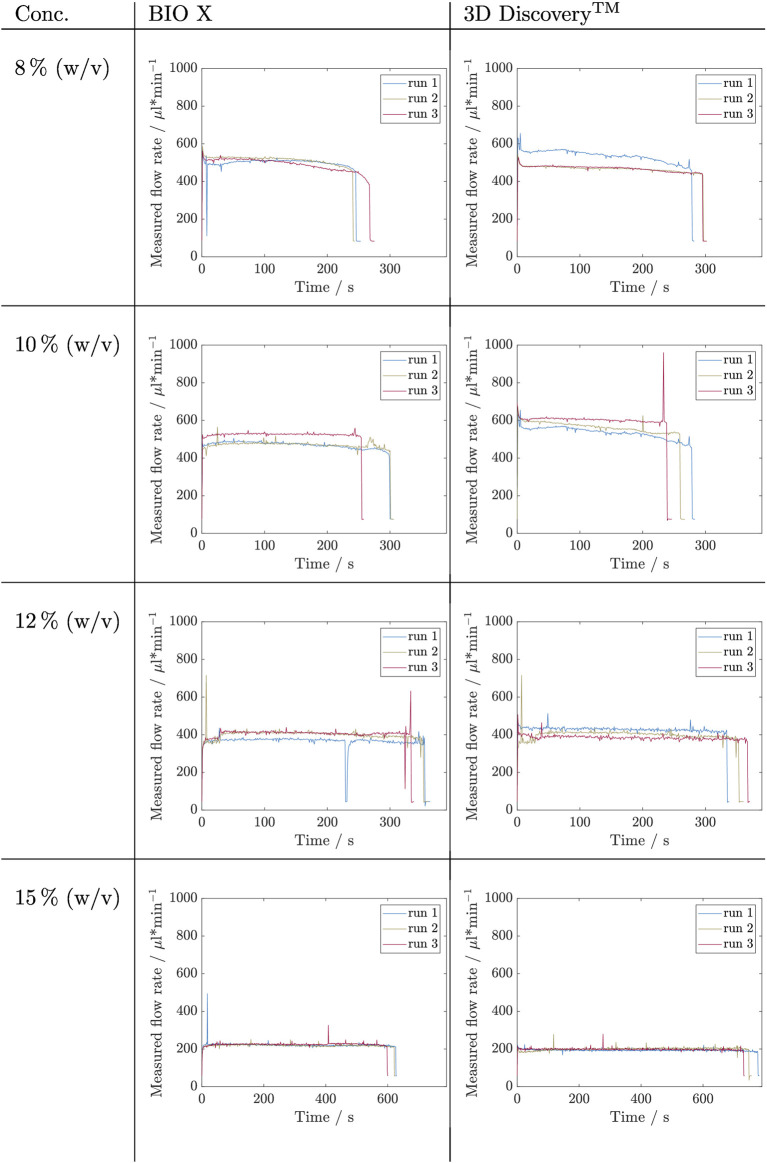
Results of filling level influence investigation for inks containing alginate concentrations of 8, 10, 12, and 15% (w/v) by measuring the flow rate during the complete emptying of a cartridge. This experiment was carried out in triplicates (*n*_*run*_ = 3) for each concentration and on two bioprinter systems, namely BIO X and 3D Discovery™. The respective set pressures are listed in Table 3.

From a process engineering point of view it is of utmost importance to assess whether dynamic changes within the system i.e. the bioink filling level within a cartridge has an impact on the extrusion flow and thus on the whole bioprinting process. Therefore, cartridges were filled up to the same level with 3 ml of ink containing different concentrations of Kolliphor or alginate. Then, a constant pressure was applied to the cartridge and the flow rate was monitored by the flow sensor. The set pressure was different depending on the ink, but identical in each case for the two printing systems used (see Table 2). This was done for all concentrations in triplicates (*n*_*run*_ = 3). The results are depicted in Figures 5, 6. The flow rate is shown over time during which the cartridge was emptied. On the left side are the flow rates that were measured for the BIO X and on the right side data from the 3D Discovery™ for comparison. The course of each experiment can be divided qualitatively into three phases: P1 - start-up, P2 - constant flowrate, P3 - flow rate drop.

From a first glance at the Kolliphor ink runs, it is noticable that for both printing systems data of 20% (w/v) and 25% (w/v) are noisier compared to data of solutions with 15% (w/v) Kolliphor. All runs with 15% (w/v) Kolliphor solution show using the BIO X printer an initial peak (P1), which then falls to a relatively constant flow rate between 2,500 and 2,800 µL min^−1^ (P2) and then drops for each run differently (P3). P3 sets in latest at run 3. In comparison, the 3D Discovery™ shows less distinct initial peaks during P1 and run 1 reaches a constant flowrate during P2. The flow rate of the other two runs falls permanently and inconsistently. In P3, the drops are inconsistent and thus exhibit different extrusion rates. A comparison of the two systems shows that the BIO X achieves higher flow rates with maximum values in P2 between 2,500 and 2,800 µL min^−1^ compared to the flow rates at 3D Discovery™, which are between 1,300 and 1700 µL min^−1^ at the most. Accordingly, the cartridges for the BIO X are empty after 90–120 s and for 3D Discovery™ later after 155–210 s. For 20% (w/v) Kolliphor solution the earlier observed characteristic peak in P1 could not be observed and the extrusion process started directly in P2. Both, BIO X with a decreasing flow rate from approximately 330 µL min^−1^ to 270 µL min^−1^ and the 3D Discovery™ with flow rates in range of 200–260 µL min^−1^, show no stable flow rate in P2 and the runs are not comparable. During P1 and the beginning of P2 of the 25% (w/v) Kolliphor solution, the maximum measurable flow rate was exceeded for both bioprinters and therefore the values are partially truncated at the top. Thus a clear statement on the development of P1 can not be made. The BIO X runs in fluctuate strongly in P1. No clear trend is discernible, and the flow rates only stabilize after approximately 250 s in P2. The level of the flow rate of the individual runs in P2 differs and is in a range of 250–350 µL min^−1^. In P2 of the 3D Discovery™ runs, all three runs have a flow rate of 300–350 µL min^−1^ after 150 s, whereby run 3 continuously drops from 400 to 260 µL min^−1^ and does not reach a stable phase.

The data obtained for the alginate inks is not as noisy as that of Kolliphor inks, however, the noise again increases with increasing alginate concentration. No initial peak in P1 could be detected in any run. In P2 of the 8% (w/v) alginate solution, the three runs show a stable flow rate above 500 µL min^−1^ with temporary differences up to 20 µL min^−1^. Run 3 decreases constantly. Except for ink containing 15% (w/v) alginate, the same pressure on the 3D Discovery™ resulted in higher flow rates up to a factor of 1.4 during P2 for 10% (w/v) alginate ink. The flow rate curves at 15% (w/v) alginate ink are on the BIO X constant in P2 for 600 s at 220 µL min^−1^ and again slightly higher compared to the P2 on 3D Discovery™ where the flow rates are around 200 µL min^−1^. Here, run 1 decreases constantly and run 2 increases during emptying. So, again, no trend is visible.

### 3.4 Reproducibility Experiments

**FIGURE 7:**
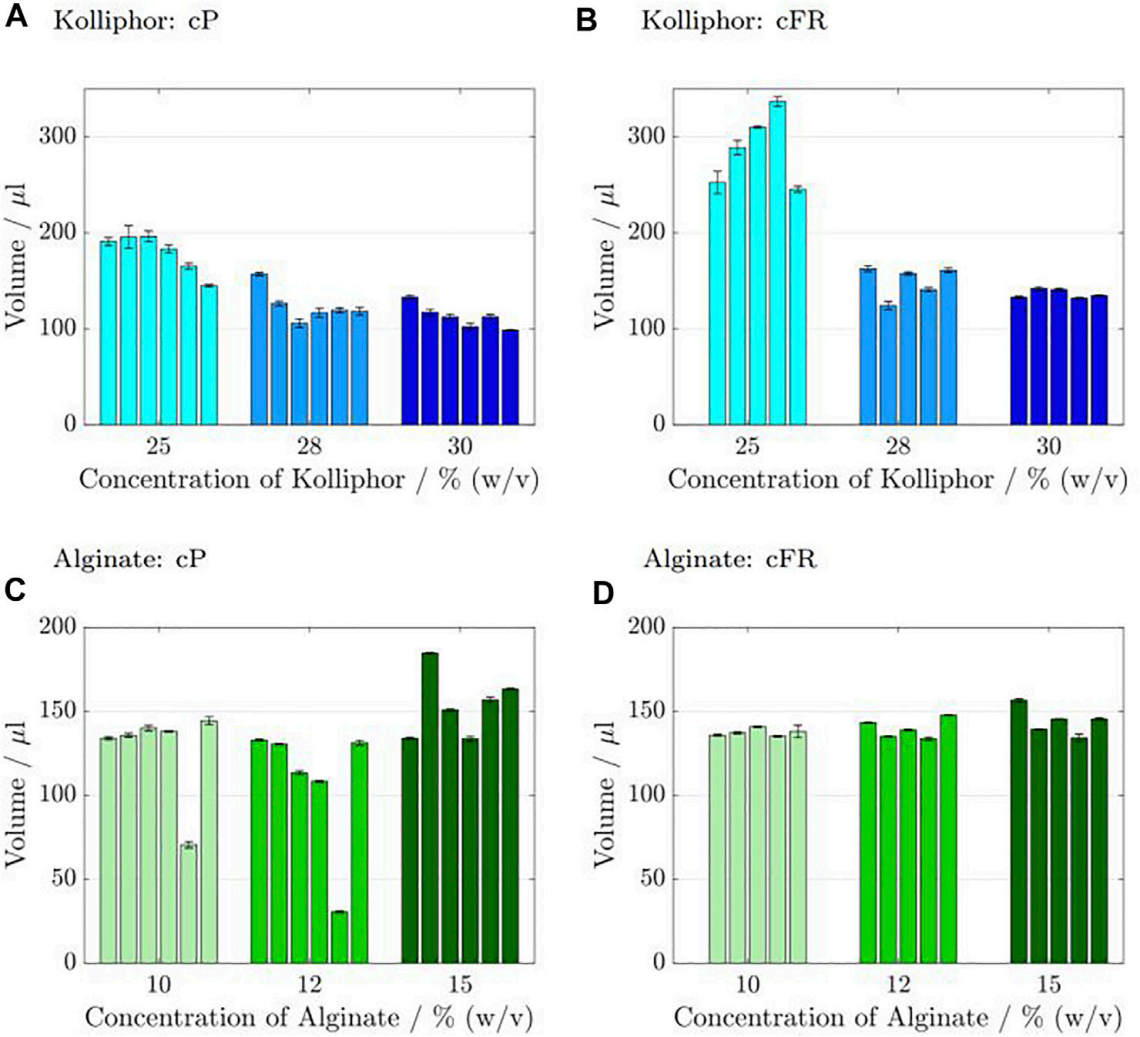
Results of the reproducibility tests for Kolliphor containing inks **(A–B)** and Alginate containing inks **(C–D)**. Six runs (*n*_*run*_ = 6) were carried out in which 3 samples (*n*_*sample*_ = 3) were printed each for the cP and the cFR approach. Consequently, 18 cylinders for the cP approach and 18 cylinders for the cFR approach were printed in total. The obtained mean values and deviations of the 3 samples belonging to one run are shown in 1 bar. The cP results where the pressure was kept constant for all six runs are presented in **(A,C)**, and the cFR results where the pressure was adapted to set a constant flow rate are shown in **(B,D)**. As the 3 samples from run 1 with cFR were used as calibration set for the flow rate determination, there is 1 bar less.

Reproducibility is a measure describing the potential of producing an object or measurement repeatedly with the same accuracy. To deliver a brief and general overview of the reproducibility for the two different process modes cP and cFR, 6 runs (*n*_*run*_ = 6) were carried out in which 3 identical cylinder samples per alginate and per Kolliphor concentration were printed (*n*_*sample*_ = 3). In the first cP approach, the same predefined pressure was used for each run (see Table 5). In the second cFR approach, the pressure was adjusted by using the flow sensor as calibration tool prior to each run to meet a predefined flow rate. The results obtained are depicted in Figure 7, where the mean volumes of the 3 samples of 1 run with standard deviation are plotted against the respective concentration.

For 25% (w/v) Kolliphor containing ink, the minimum volume at cP was 145 µL and the maximum volume was 196.18 µL. The deviation was in the range of 1.3 ± 11.95 µL during the 6 runs. In comparison, the cFR values are higher, i.e. in the range of 245.42–336.89 µL with a standard deviation of maximum ±11.79 µL during the 6 runs. The cP values with 28% (w/v) Kolliphor containing ink are lower with volumes in the range from 118.37–157.11 µL with a maximum standard deviation of 4.58 µL during the 6 runs. The cFR results are varying from 124.37–162.66 µL during the 6 runs with a maximum standard deviation of 4.09 µL. For 30% (w/v) Kolliphor containing ink with cP process strategy, volumes in the range of 98.6–133.1 µL with a deviation between 0.76 and 3.52 µL were measured during the 6 runs in comparison to the cFR strategy with volumes between 132.08 and 142.15 µL and a standard deviation of up to ±1.96 µL during the 6 runs. The cylinders printed with alginate containing ink are smaller than the cylinders printed with Kolliphor containing ink. For 10% (w/v) alginate containing ink, the cFF cylinder volumes are in the range of 70–144.4 µL with a maximum deviation of ±1.52 µL during the 6 runs, but run 5 falls out with only about half the weight compared to the others. There is no outlier for the cFR results, which are in the range of 135.15–140.86 µL with a maximum standard deviation of ±3.74 µL during the 6 runs. For the 12% (w/v) alginate containing ink cP results, the cylinders of run 5 are smaller with 30.53 ± 0.58 µL for that run in comparison to the other 5 runs which is between 113.38 and 132.82 µL with a maximum deviation of 1.23 µL. The cFR volumes using the same ink concentration are slightly higher with volumes between 133.61 and 147.74 µL ± maximum 1.5 µL during 6 runs. For 15% (w/v) alginate solutions, the range of volumes is from 133.56–163.35 µL ± maximum 1.42 µL during the 6 runs. In general, there are higher deviations between the individual runs for cP than for cFR, in which the volumes are between 134.15 and 156.61 µL with a maximum standard deviation of ±2.3 µL.

**FIGURE 8:**
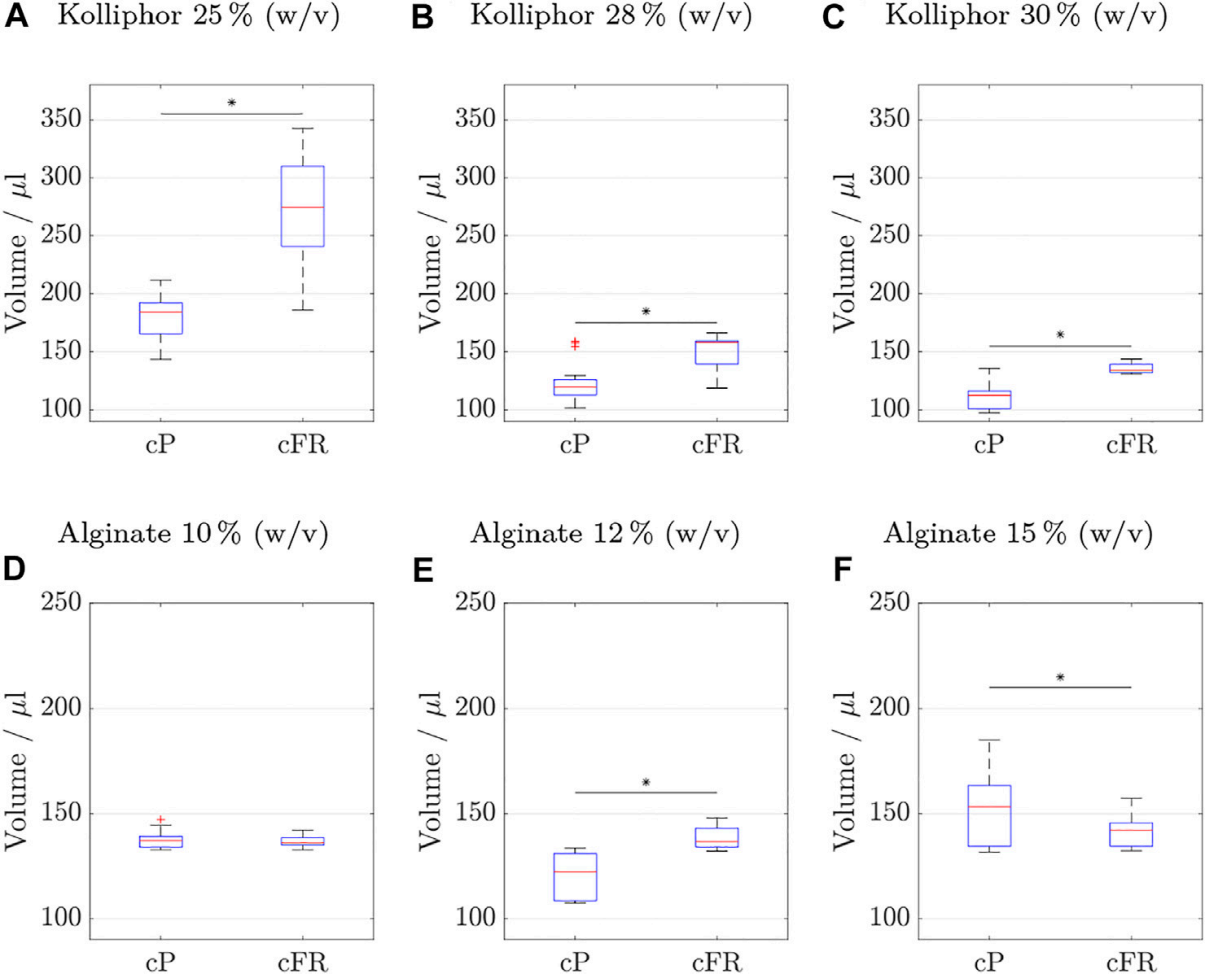
Cylinder volumes (*n*_*run*_ = 6 with *n*_*sample*_ = 3 resulting in 18 cylinders) of the reproducibility test comparing the two process strategies: on the left side using constant pressure (cP) and on the right side applying a constant flow rate (cFR). Values are considered outliers if they are more than 1.5 times the interquartile range from the bottom or top of the box. The results obtained with inks containing different Kolliphor concentrations are shown in **(A–C)** and with inks containing different alginate concentrations in **(D–F)**. Statistically significant differences between cFR and cP were found between all data sets except for ink containing 10% (w/v) alginate. The specific *p*-values are **(A)** 4e-6, **(B)** 1e-6, **(C)** 5e-6, **(D)** 2e-6, and **(F)** 0.047.

As the Anderson-Darling test did not result in a normal distribution for all data sets, a Mann Whitney U test was performed. For a better comparison and statistical evaluation of the distribution of the data sets of cP and cFR, Figure 8 visualizes the box plots of the two strategies for each ink concentration. The cylinders of every ink from all runs were summarized in one box plot and cP was compared with cFR.

Statistically significant differences between the two process strategies were found for all data sets, except for ink containing 10% (w/v) alginate. To make the comparison easier, key figures for the box plots are listed for Kolliphor containing inks in Table 6 and for alginate containing inks in Table 7.

**TABLE 6:** Boxplot key figures of Kolliphor containing inks boxplots shown in Figures 8A–C.

Ink_*Kolliphor*_	Constant pressure (cP)			Constant flow rate (cFR)		
[% (w/v)]	25	28	30	25	28	30
Min. volume [µl]	143.48	101.5	97.57	185.9	118.82	130.96
Max. volume [µl]	211.6	158.94	135.66	342.72	166.35	143.81
Range [µl]	68.12	57.44	38.1	156.81	47.53	12.86
Median	184.4	119.9	112.59	274.51	158.08	134.27
Lower quartile	165.51	112.95	101.12	240.88	139.7	132.21
Upper quartile	192.28	126.13	116.38	309.97	159.42	139.5
Variance	406.41	283.72	138.47	2e+3	206.45	19.71
Standard deviation	20.16	16.84	11.77	49.45	14.37	4.44
Coefficient of variance	0.11	0.14	0.1	0.18	0.1	0.03
Interquartile distance	26.76	13.18	15.25	69.1	19.72	7.29

**TABLE 7:** Boxplot key figures of alginate containing boxplots shown in Figures 8D–F.

Ink_*Alginate*_	Constant pressure (cP)			Constant flow rate (cFR)		
[% (w/v)]	10	12	15	10	12	15
Min. volume [µl]	67.83	29.87	131.68	132.79	132.19	132.34
Max. volume [µl]	147.14	133.52	185.13	142.16	147.93	157.33
Range [µl]	79.31	103.65	53.44	9.38	15.74	24.99
Median	137.24	122.37	153.21	136.14	136.74	142.17
Lower quartile	133.94	108.58	134.4	134.99	134.09	134.4
Upper quartile	139.2	130.96	163.41	138.53	143.1	145.63
Variance	692.59	1e+3	330.92	8.1	31.4	66.6
Standard deviation	26.32	36.85	18.2	2.85	5.6	8.16
Coefficient of variance	0.2	0.34	0.11	0.02	0.04	0.06
Interquartile distance	5.26	22.38	29.01	3.54	9.01	11.23

In the following, only the coefficient of variance is discussed for the sake of clarity. The advantage is that outliers do not have such a strong influence, as with the range and the data distribution is considered more than if only the mean values were considered. The advantage is that the standard deviation is considered in relation to the mean value. The coefficient of variance increased by 63% for 25% (w/v) Kolliphor ink. For 28 and 30% (w/v) Kolliphor ink, the coefficient of variance decreased by 29 and 70%, respectively.

For alginate containing inks, the results are even clearer and the coefficient of variance drops at least 46% for 15% (w/v) alginate ink when printed by a constant flow rate. For a 10% (w/v) alginate ink, the coefficient of variance was reduced by 90%.

## 4 Discussion

Progress in the field of bioprinting has been made, but the shift from research to market is still far from being complete. This study served to evaluate whether the reproducibility of bioprinting processes is improved using a set flow rate as a process parameter, since robust and reliable processes are a basic requirement for medical applications. Already in another study it is concluded that extrusion based bioprinting process is affected by bioink and process-related influences which again can result in a low reproducibility (Kesti et al., 2016). To investigate how reproducibility can be increased in pneumatic systems a flow sensor was incorporated into the printing systems and calibrated for appropriate ink compositions. The deviation of the sensor data from the weighed data is acceptable for Kolliphor inks being below 10% for all concentrations. The standard deviations at inks containing 20% (w/v) and 25% (w/v) Kolliphor are relatively high when compared to the other systems analyzed. A possible explanation is the sol-gel transition temperature which is closer to room temperature at 20–25% (w/v) Kolliphor than for the other examined concentrations (Dumortier et al., 2006). Here, the applied sensor may have problems to measure gels as it was developed and optimized for liquids. Likewise, no deviation greater than 10% was measured for alginate inks, except for ink containing 15% (w/v) alginate with a deviation of 16%. The obtained standard deviations for alginate inks are much smaller than those containing Kolliphor. This said, both inks show a correlation between deviations obtained and ink concentration applied. A reason might be that the viscosity increase decreases the accuracy of the sensor. The sensor used in this study, employs a thermal principle which means that temperature changes impact the sensor output and network inhomogeneities can result in different heat conduction coefficients, which impair the measuring accuracy. Local inhomogeneities also have an influence on the material flow (Fisch et al., 1101; Wang et al., 1994; Seiffert and Sprakel, 2012). Taken together, inhomogeneities might lead to both, fluctuating measurements and to unsteady flow. On the basis of the data, no discrimination is possible to what extent the two effects lead to noisy data. However, in conclusion, sensor performance was considered sufficient and was used for further experiments. What is also becoming apparent is that each ink composition needs different pressures and behaves differently. An automated setting of the pressure at a fixed flow rate would be of great advantage here (see behaviour of different inks in Section 3.2).

In a following step, the influence of the filling level inside the cartridge on the flow rate was examined with the aid of the sensor using a constant pressure setup (cP) for all concentrations at two bioprinters, namely BIO X and 3D Discovery™. The flow rate during a complete emptying—until no ink was extruded anymore—of a cartridge was recorded in triplicates for different alginate and Kolliphor concentrations. Here, again, it is generally noticeable that higher viscosity inks lead to noisy data. The data, however, could not be averaged because the runs were not comparable. The Kolliphor time courses of the flow rate in particular differ greatly and hardly any stable areas could be specified. Alginate as a polymer solution has shown more reproducible processes, but again exceptions with a steady drop of the flow rate were experienced. It is particularly noticeable that the same pressure setup resulted in different flow rates in different bioprinter systems. The same parameters on the 3D Discovery™ resulted in lower flow rates for Kolliphor and higher flow rates for alginate. Thus, there is no trend, and a simple inter-system transferability is not given. These results confirm that the flow rates can vary depending on the materials used during a printing process and that constant control of the flow rate may improve printing results by ensuring a steady flow rate (see data on filling level in Section 3.3).

In order to investigate whether an extrusion process based on flow rates leads to an increase in reproducibility, three cylinder samples were printed during 6 runs with two different process controls. In the constant pressure approach (cP) in every run the same pressure was applied. This is in accordance with the common procedure given by system manufacturers. In the other cFR approach, the pressure was adapted to obtain a set flow rate which was verified with the flow sensor as calibration tool. The results indicate that the run-to-run deviations were inconsistent and rather high for the cP process mode, while the standard deviation within one run is quite low. One reason for this might be that environmental conditions fluctuate strongly between days and printig sessions, but only marginally during the relatively short duration of the printing session itself. This requires that the pressure or process parameters need to be adjusted prior to each run or printing session (see behaviour over 6 runs in Section 3.4). This said, considering the high deviations in flow rate as a function of cartridge filling level, continuous pressure adjustment would be necessary for longer printing processes. Except for ink containing 25% (w/v) Kolliphor, the standard deviation and coefficient of variance could be improved by a calibration before each run. The review of the sensor performance already showed the measurement problems of the sensor for ink containing 25% (w/v) Kolliphor which can be explained by the sol-gel-transition temperature of Kolliphor close to room temperature (Dumortier et al., 2006). As can be seen from the fluctuations of flow during the runs examining the influence of the cartridge filling level, the noisiness of the data increases with higher polymer concentration and it becomes increasingly difficult to set the flow rate precisely. However, to put it all in a nutshell, it could be shown that a flow rate-based cFR principle leads to more comparable and more reproducible results. Of course, research work is necessary to implement the principle on bioprinters and to construct bioprinters with flow sensors which have been developed and appropriated for inks or rather bioinks. It is beneficial to control the pneumatic extrusion as changes in the bioink viscosity results in flow inhomogeneities which do not allow a reproducible extrusion of filament (Kesti et al., 2016). Other mechanical extrusion systems which are screw or piston driven promise a higher spatial control and constant flow rates as no gas volume is compressed before. Compared to the pneumatic systems, they are more complex with more components and are not as widespread (Murphy and Atala, 2014b). They are able to extrude higher viscosity materials, but large driving forces can cause damage to cell walls (Ning and Chen, 2017; Ning et al., 2020). During the construction, problems with the pneumatic transport regime must be taken into account because the concentration and velocity of bioinks is sometimes network inhomogeneous (Barratt et al., 2000). In the future, a distinction must then also be made between two process controls. Calibration directly before the printing process allows a reaction to daily environmental fluctuations, changes of printer systems, and changes of bioinks. On the other hand, a continuous pressure control would allow an adjustment of the pressure, which may be necessary due to temperature changes already during the printing process, changes of the cartridge filling level, larger inhomogeneities, and nozzle clogging.

## 5 Conclusion

Reproducible and robust processes are necessary to make the leap from reasearch to medical application. We demonstrated that employing a flow rate-based extrusion process can reduce the variations between printed objects and increase the reproducibility of bioprinting applications.

In preliminary tests, the sensor used was found to be suitable for the measurement of bioinks. Furthermore, it has been demonstrated that the filling level in the cartridge and the printer type have an influence on the flow rate. It was shown that the cFR approach led to a higher reproducibility than the cP approachd as it was possible to respond well to variations in environmental conditions between different runs and printing sessions. An automated calibration for automatic pressure determination for a defined flow rate would be desirable. Even better would be automated pressure readjustment in a feed back loop to keep a flow rate constant. This would turn the static monitoring of the flow rate in a dynamic, adaptable proces and variations such as cartridge filling level and inhomogeneities can be responded to directly. In addition, the sensor must be adapted to the respective viscosity ranges of the bioinks and the sensor should also be compatible for the respective temperature range. Bioinks are sometimes printed at different temperatures and the temperature has an influence both on the rheological properties of the bioink and on the thermally based measuring principle of the sensor. But another measuring principle would also be conceivable. In summary, the experiments provided a proof of concept for the flow rate-based process management to increase reproducibility and this must now be integrated into the bioprinters.

## Data Availability

The original contributions presented in the study are included in the article/Supplementary Material, further inquiries can be directed to the corresponding author.
