# Supplementary material for: Evaluation of the Reproducibility and Robustness of Extrusion-Based Bioprinting Processes Applying a Flow Sensor
Source: Front Bioeng Biotechnol. 2022 Mar 3;10:831350. doi: 10.3389/fbioe.2022.831350 (PMC8927775; doi:10.3389/fbioe.2022.831350)
Supplement: Supplementary file 1 [file DataSheet1.PDF]

Table 1: Results of the density calibration for sodium alginate and Kolliphor using a micro liquid density sensor. For sodium alginate the concentrations of 0.25, 0.5, 0.75, 1, 1.5, 2, 2.5, 3 % (w/v) and Kolliphor concentrations of 1, 2, 3, 5, 10, 15 % (w/v) were measured in triplicate.

**Hydrogel**	**Density calibration graph**
Sodium alginate	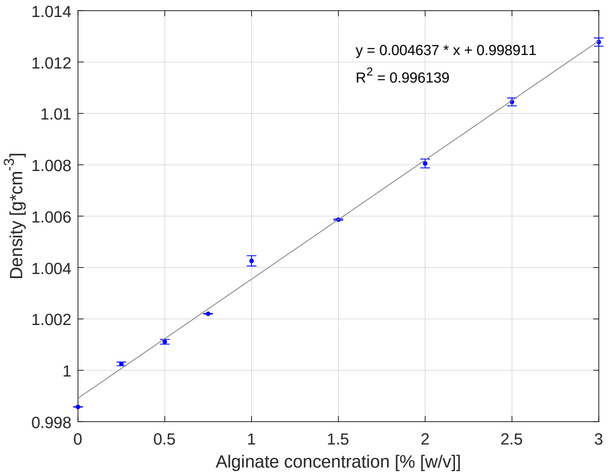
Kolliphor P 407	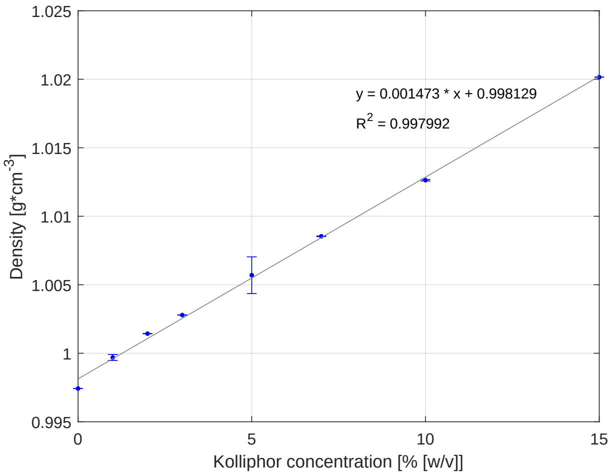

Table 2: Results of the flow sensor SLI-1000 FMK calibration for alginate concentrations of 8, 10, 12 and 15 % (w/v) and for Kolliphor concentrations of 15, 20, 25, 28 and 30 % (w/v). The flow sensor was connected via luer lock to a syringe pump and each flow rate was measured in triplicate.

**Hydrogel and concentration**	**Flow sensor SLI-1000 FMK calibration graph**
8 % (w/v) sodium alginate	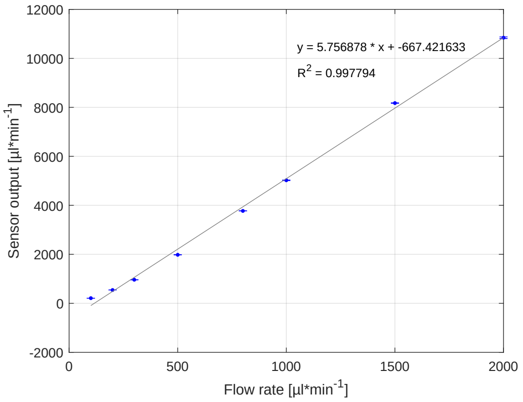
10 % (w/v) sodium alginate	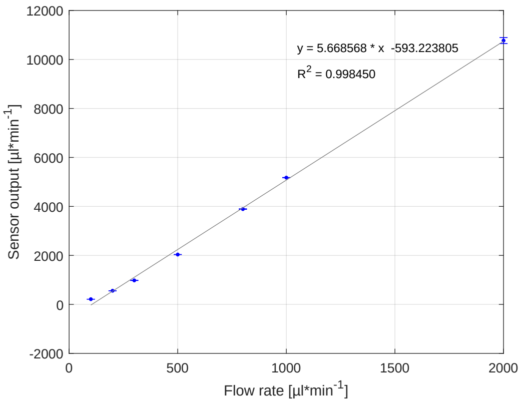

12 % (w/v) sodium alginate

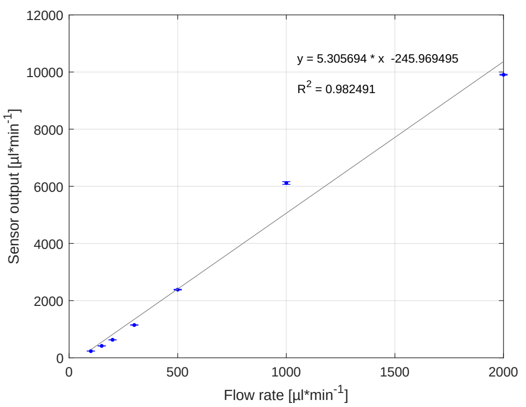

15 % (w/v) sodium alginate

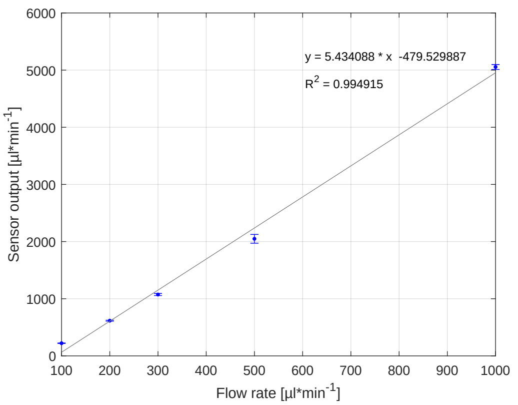

15 % (w/v) kolliphor P 407

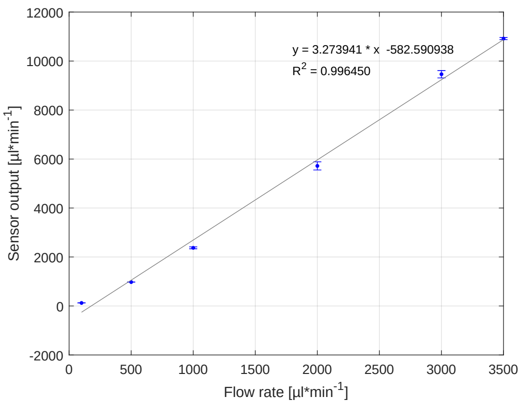

20 % (w/v) kolliphor P 407	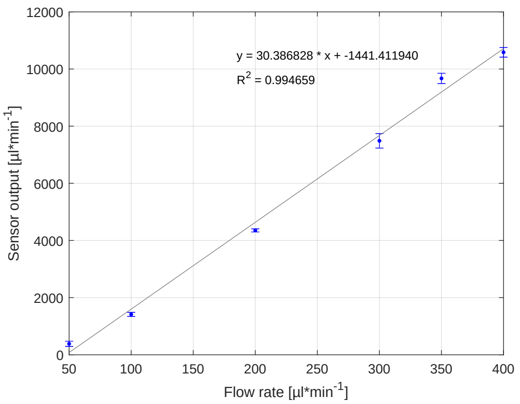
25 % (w/v) kolliphor P 407	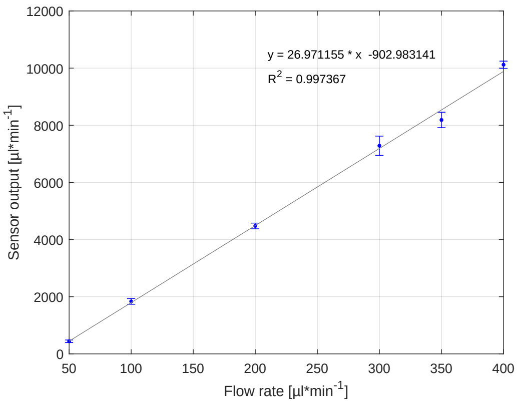
28 % (w/v) kolliphor P 407	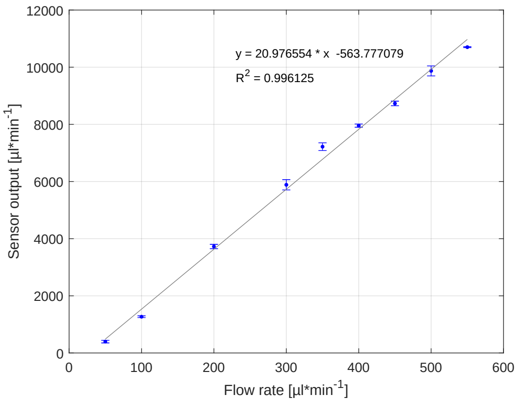

30 % (w/v) kolliphor P 407	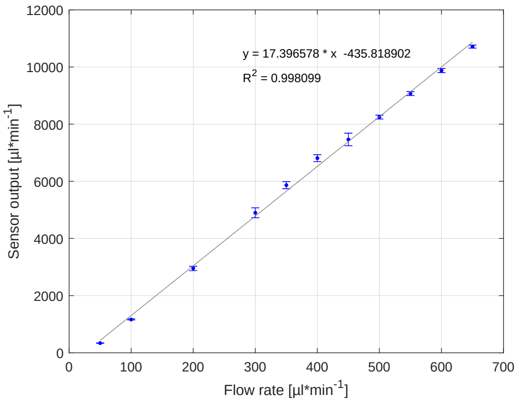
